# Non-Covalent Interactions of Lotus Root Polysaccharides and Polyphenols and their Regulatory Mechanism on Macrophage Functions

**DOI:** 10.3390/foods13223543

**Published:** 2024-11-06

**Authors:** Yajie Li, Nan Huang, Qiulan Liu, Ying Sun, Kaidi Peng, Xueyu Jiang, Yang Yi

**Affiliations:** Hubei Key Laboratory for Processing and Transformation of Agricultural Products, College of Food Science and Engineering, Wuhan Polytechnic University, Wuhan 430023, China; liyjie58@163.com (Y.L.); hyysgxnn@163.com (N.H.); liuqiulan575@163.com (Q.L.); sunying7535@163.com (Y.S.); kaidi19930218@outlook.com (K.P.); yiy86@whpu.edu.cn (Y.Y.)

**Keywords:** lotus root polysaccharide, polyphenol, noncovalent interaction, immunomodulation, antioxidant

## Abstract

Despite the interaction between polyphenols and polysaccharides in food products, their specific non-covalent interactions and effects on macrophage functions are not well understood. Therefore, the interaction and mechanism of purified lotus root polysaccharide (PLRP) with polyphenols, and the regulatory mechanisms of the PLRP-polyphenol complex on the macrophage functionals were studied. By combining ferulic acid (FA) and chlorogenic acid (CHA) with PLRP, the complexes PLRP-FA, PLRP-CHA and the physical mixtures PLRP&FA and PLRP&CHA were prepared, where their mass ratios of polyphenols to PLRP were 143.97 and 601.67 mg g^−1^. Nuclear magnetic resonance (NMR), Fourier-transform infrared (FTIR), Ultraviolet (UV), and Transmission electron microscopy (TEM) analyses confirmed that PLRP and polyphenols may engage in non-covalent interactions via hydrogen bonds and hydrophobic interactions. We confirmed that non-covalent interactions led to high molecular weight, dense complexes. Both PLRP and its polyphenol complexes stimulated NO production by macrophages to varying degrees without exacerbating lipopolysaccharide-induced inflammatory responses. PLRP and PLRP-polyphenol complexes repaired cells with impaired antioxidant capacity, depending on doses. Those results indicated that after the combination of lotus root polysaccharide and polyphenol, the molecular weight and conformation changed significantly, which influenced the biological activity. RNA-seq analysis suggested that the regulatory mechanism of PLRP-polyphenol complex in macrophages may mainly involve oxidative phosphorylation, FoxO, TNF, IL-17, MAPK, NF-kappa B, and other signaling pathways. This study investigated the effects of polyphenol binding on the physicochemical characteristics and functional activities of polysaccharides, which provided references for the development of polysaccharide functional products and the control of nutritional quality.

## 1. Introduction

Polyphenols and polysaccharides are coexisting nutritional food components that can undergo non-covalent interactions during food processing and consumption when their inherent structural arrangements are disrupted. These non-covalent interactions typically involve specific bioactive small molecules that bind to biomacromolecules and form specific and reversible complexes. Polyphenol–polysaccharide complexes in foods mainly coalesce through noncovalent interactions [1]. With noncovalent interactions, polyphenols can be retained via surface adsorption [2] or encapsulation by polysaccharides when hydrophobic cavities form through the chain alignment of polysaccharides [3]. Molecular forces, including hydrogen bonds, hydrophobic interactions, Van der Waals forces, and electrostatic interactions, are involved in the physical complex formation [4,5]. Currently, the methods for studying such interactions include ultraviolet-visible (UV-Vis) spectroscopy, Fourier-transform infrared (FTIR) spectroscopy, high-performance size-exclusion chromatography (HPSEC), nuclear magnetic resonance (NMR) spectroscopy, and microscopic observations. For instance, Li et al. inferred the chemical binding between soluble dietary fiber (SDF) and phenols using UV-Vis and FTIR spectroscopy [6]. Wu et al. investigated the binding of tea polyphenols to β-glucans using NMR analysis and found that hydrogen bonds primarily contributed to their binding [7]. Additionally, atomic-force microscopy was used to observe the microstructure of lotus rhizome SDF and its complexes, revealing that non-covalent binding might increase the rigidity of SDF molecules, thereby reducing molecular entanglement [8]. Scanning electron microscopy (SEM) was employed to observe the surface morphology of corn silk polysaccharides, revealing strong intermolecular interactions [9]. Previous findings have indicated that non-covalent interactions between lotus root polysaccharides (LRP), ferulic acid (FA), and chlorogenic acid (CHA) can lead to complex formations [10]. Previous research suggested that the non-covalent interactions between lotus rhizome polysaccharides and polyphenols may involve hydrogen bonds, electrostatic interactions, and hydrophobic interactions, with variations in polyphenol types [11]. However, the specific mechanisms underlying these non-covalent interactions remain unclear. Therefore, studying the mechanism of LRP-polyphenol complex formation is important. In this study, we used anion-exchange resin 717 to further purify lotus rhizome polysaccharides and prepare PLRP-polyphenol complexes. Various analytical methods, including spectroscopy, chromatography, spectroscopy, and microscopic observations, have been employed to analyze the structural and microstructural differences between purified polysaccharides and their polyphenol complexes to elucidate the molecular mechanisms underlying complex formation.

Macrophages, a component of the immune system, have the capability to eliminate pathogens through phagocytosis during nonspecific immune responses. Additionally, macrophages are capable of releasing cytokines and chemokines, mediating local inflammations, and recruiting various immune cells to infection sites to eliminate pathogens [12]. During the inflammation, macrophages (through lipopolysaccharides [LPS] and recognition receptors such as Toll-like receptor 4), can activate downstream signaling pathways, typically involving nuclear factor-κB (NF-κB) and mitogen-activated protein kinases (MAPKs). Activated signals linked to inflammation may trigger the transcription and translation of a series of pro-inflammatory factors, including inducible nitric oxide (NO) synthase, cyclooxygenase-2 (COX2), tumor necrosis factor alpha (TNF-α), and interleukin-6 (IL-6). Moreover, they played a crucial role in the development of inflammatory diseases [13], thus significantly influencing both innate and acquired immunity. Numerous research findings indicate that polysaccharides are crucial in boosting the immune and antioxidant activities of macrophages [14].

Previous findings have indicated that lotus rhizome polysaccharides and their polyphenol complexes exhibit certain antioxidant and macrophage function-regulating capabilities [15]. However, the specific regulatory mechanisms underlying these processes remain unclear. Here, we aimed to evaluate, for the first time, the in vitro immunomodulatory and antioxidant activities of lotus rhizome polysaccharides and their polyphenol complexes using a macrophage model. In addition, we used RNA sequencing (RNA-seq) to systematically explore the key genes and signaling pathways involved in regulating macrophage function.

## 2. Materials and Methods

### 2.1. Materials and Chemicals

Fresh lotus root (cultivar: Elian NO.5) was obtained from Wuhan Jinshui-qiliang Agricultural Products Co., Ltd. (Wuhan, China). Ferulic acid (FA, > 95%) and Chlorogenic acid (CHA, 98%) were purchased from Aladdin Co., Ltd. (Shanghai, China). Folin-Ciocalteu (Folin, >99%) reagent and other analytical reagents were purchased from Singpharm Chemical Reagent Co., Ltd. (Shanghai, China). All other reagents were of analytical grade and used without further purification. The cells used in our experiments were purchased from Procell Life Science & Technology Co., Ltd. (Wuhan, China). Accession Numbers: CL-0190, Database Name: RAW 264.0.

### 2.2. Purification of LRP

Lotus rhizome polysaccharides (LRP) was extracted from lotus root and further purified by removing the protein, CaCO_3_ and impurities according our previous method [16]. The purified lotus rhizome polysaccharide (PLRP) fraction was obtained after dialysis and freeze-drying, with a polysaccharide content of 90.08%. The specific process method was shown in Figure S1.

### 2.3. Preparation of PLRP-Polyphenol Complexes

PLRP-polyphenol complexes were prepared according to our previously described method with minor modifications [16]. Solutions of PLRP (2 mg mL^−1^), FA (1 mg mL^−1^), and CHA (1 mg mL^−1^) were mixed under specific conditions, as shown in Table S1 and Figure S2. PLRP-polyphenol complexes were freeze-dried and named PLRP-FA and PLRP-CHA. Additionally, the physical mixtures were directly mixed with PLRP and polyphenols according to the polyphenols content, named PLRP&FA and PLRP&CHA, respectively.

### 2.4. Characterizations of PLRP-Polyphenol Complexes

UV–Vis spectra were recorded using a UV–Vis spectrophotometer (TU-1810, Puxi, China). FTIR spectra were obtained using an FTIR spectrometer (NEXUS670, Nicolet, Madison, WI, USA). 1H NMR spectra were acquired on an Avance-III 400 MHz spectrometer (Bruker, Billerica, MA, USA). SEM images were recorded using a HITACHI 5-4800 microscope (Hitachi, Chiyoda, Japan). Transmission electron microscopy (TEM) images were observed on a JEM-2010 (HT) electron microscope (JEOL, Tokyo, Japan). The molecular-weight (Mw) distribution of the samples was measured using the HPSEC equipped with multi-angle laser light scattering and refractive index (RI) detection (Agilent Technologies, Santa Clara, CA, USA).

### 2.5. Evaluation of Macrophages Immunomodulation

#### 2.5.1. Measurement of Cell Proliferation Rates

200 μL of macrophage suspension (2 × 10^5^ cells mL^−1^) was seeded with samples at different concentrations (50, 100, 200 or 400 μg mL^−1^), while another set received only blank medium (200 μL). Subsequently, CCK-8 reagent was added after incubation for 24 h. After another 2 h of incubation, the absorbance at 450 nm was measured.

#### 2.5.2. Determination of NO Production by Macrophages

NO production was measured according to the method described by Yi et al. [17], with slight modification. Briefly, after macrophages were cultured, 500 μL of culture medium containing sample (50, 100, or 200 μg mL^−1^) and LPS (500 mg mL^−1^) without fetal bovine serum was added. A blank control group without stimuli and an LPS control group (500 mg mL^−1^) were also designed for each group as parallel wells. NO levels were measured in the supernatants at 24 h and 48 h after culture.

### 2.6. Evaluation of Macrophage Antioxidant Activity

#### 2.6.1. Establishment of Macrophage Oxidative Damage Model

Cultured macrophages were adjusted to a density of 2.0 × 10^5^ cells mL^−1^, and 200 μL of the cell suspension was added to incubate for 24 h at 37 °C. Then medium containing different concentrations of H_2_O_2_ solution was added to each well. Subsequently, an MTT reagent was added, and the absorbance at 490 nm was measured. The cell viability was calculated using Equation (1):

(1) $$Cell\;Viability\;\left(\%\right)=\left(1-\frac{A_{0}-A_{1}}{A_{0}}\right)\times 100$$

where *A_0_* represents the average absorbance of the blank group, and *A_1_* represents the average absorbance of the groups exposed to different H_2_O_2_ concentrations.

#### 2.6.2. Determination of SOD, MDA, and T-AOC Levels in Cells

Macrophages (2.0 × 10^5^ cells mL^−1^) were seeded, and the supernatant was replaced with medium containing with or without H_2_O_2_ (50, 100, or 200 μmol L^−1^). Cells in the damage groups were pre-treated with H_2_O_2_ for 30 min. Subsequently, 100 μmol L^−1^ H_2_O_2_ was added to both the control and damage groups and the cells were incubated for an additional 24 h. After that, the cells were sonicated in an ice-water bath. The levels of SOD, T-AOC, and MDA in both the cells and culture medium were measured. After treatment, the MDA was detected by the TBA quantification Kit. The supernatant of treated cells mixed with reaction liquid was reacted at 95 °C for 40 min, cooled, centrifuged, and measured at 532 nm to calculate the content of MDA. The Xanthine oxidase method was utilized to determine SOD activity. The T-AOC content was determined via the ABTS enzyme catalysis method.

### 2.7. RNA-Seq Transcriptome Sequencing

Macrophages in the damage groups were pre-treated for 30 min with 200 μg mL^−1^ H_2_O_2_ (model group). Then, 100 μmol L^−1^ H_2_O_2_ was added to both the control and model groups and the cells were incubated for an additional 24 h. After incubation, the cells were collected via centrifugation. Macrophages from the control group, model group, and PLRP, PLRP-FA, and PLRP-CHA groups were prepared for RNA sequencing. Subsequently, RNA-seq is shown in Figure S3. After filtering, the clean reads were stored as in FASTQ format using SOAPnuke-v1.5.2 software. The NOISeq method was used to screen differentially expressed genes (DEGs) between the treatment groups, using |log2 fold-change [FC]| ≥ 1 and *p* < 0.01 as screening criteria. We employed DAVID 6.8 to perform gene ontology (GO) functional classification and pathway enrichment analysis of DEGs.

### 2.8. Statistical Analysis

All data analyses were conducted using IBM SPSS Statistics 19 (Chicago, IL, USA) and quantitative data were presented as mean ± standard deviation (SD) of at least three experiments with similar results. Results were used by Duncan (D) to analyze inter-group data, *p* < 0.05 was accepted as statistical significance.

## 3. Results

### 3.1. Non-Covalent LRP-Polyphenol Interactions

**Figure 1:**
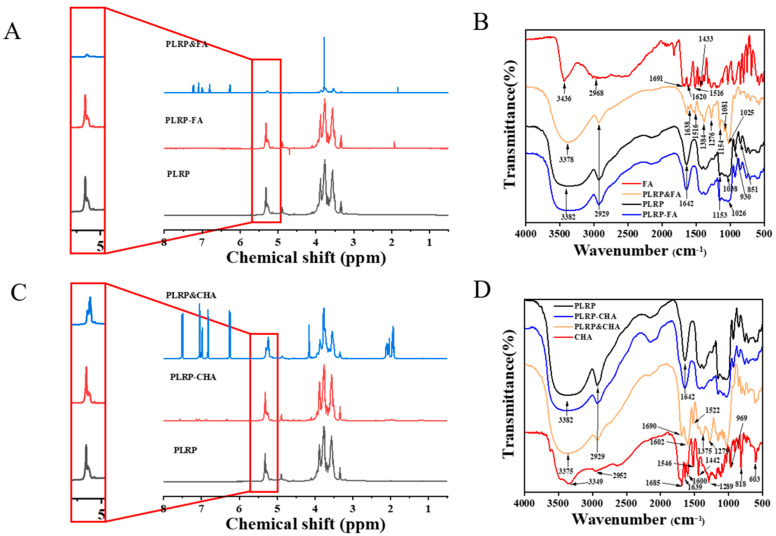
(**A**,**C**) ^1^H NMR spectra, (**B**,**D**) FTIR spectrogram of the PLRP and PLRP−polyphenol complex.

PLRP-Polyphenol complexes were prepared, with 143.97 mg of polyphenol binding to 1 g of PLRP-FA and the 601.67 mg polyphenol binding to 1 g of PLRP-CHA. As shown in Figure 1A,C, ^1^H NMR spectra were used to analyze the structures of PLRP, PLRP-polyphenol complexes, and mixtures. The α-pyranose H-1 protons had chemical shift (δ) values greater than 4.95 ppm, whereas β-pyranose H-1 protons had δ values less than 4.95 ppm, enabling the determination of sugar ring configurations. The ^1^H NMR spectrum of PLRP revealed six strong δ peaks in this region, indicating the presence of six anomeric hydrogen signals with δ values of 4.89, 4.96, 5.15, 5.26, 5.28, and 5.32 ppm, suggesting that PLRP exists in both α and β configurations.

The ^1^H NMR of FA shows δ values in the range of 7.45–7.48 ppm, which were attributed to hydrogen atoms close to the benzene ring (-CH=C). δ values in the 6.76–7.26 ppm range could be attributed to hydrogen atoms on the benzene ring; those in the 6.33–6.35 ppm range could be attributed to hydrogen atoms in C=CH- groups close to the carboxyl group; and the shift at 3.79 ppm can be attributed to the chemical shift of hydrogen atoms in -OCH_3_ groups. Similarly, CHA exhibited multiple absorption peaks in the range of 6.00–8.00 ppm, corresponding to protons at different positions on the phenolic ring. The δ peaks mentioned above were all present in PLRP&FA and PLRP&CHA complexes but were significantly weakened in PLRP-CHA complexes, and proton signals were not observed in PLRP-FA. Additionally, the mixture of PLRP with polyphenols showed peak-intensity changes and fewer peaks in the 5.22–5.32 ppm region than the PLRP complexes, indicating that non-covalent interactions occurred in this region in an aqueous system. Different types of polyphenols appear to affect the binding mechanism of PLRP to polyphenols, thereby affecting the structures of the PLRP-polyphenol complexes.

IR spectroscopy offers insights into the structural information on interactions between polysaccharides and polyphenols [18]. As shown in Figure 1B,D, PLRP exhibits typical absorption peaks characteristic of polysaccharides like LRP [19], including the O−H stretching vibration peak at 3382 cm^−1^, the C−H stretching vibration peak at 2929 cm^−1^, and the C=O stretching vibration peak at 1642 cm^−1^. Among them, the O−H stretching bands of PLRP-FA and LRP-CHA at 3382 cm^−1^ were wider than that of the mixture. A previous report [20] showed that the O−H stretching band is a sensitive indicator of hydrogen bond strengths. The wavelength and intensity of the O−H stretching band change significantly as hydrogen bonds form, and the hydrogen bond strength often increases with the widening of the O−H stretching band.

Moreover, the PLRP&FA and PLRP&CHA mixtures showed the characteristic absorption peaks of the polyphenols of FA and CHA in the wavelength range of 1630~1516 cm^−1^, respectively at 1638, 1600, 1516, 1602, and 1522 cm^−1^, which were attributed to stretching vibrations in the aromatic rings [21]. In contrast, these characteristic peaks became weakened or even disappeared in the corresponding complexes. The peaks at 1154 and 603 cm^−1^ could be attributed to the flexural-stretching vibration of phenolic hydroxyl groups [22] and the C-H bond bending of aromatic compounds [23], respectively. The peaks at these wavelengths were markedly lower in the PLRP-polyphenol complex than in the corresponding mixture. The absorption intensity of peaks at 969 and 818 cm^−1^ were also correspondingly attenuated in the complex. The attenuation of the phenolic hydroxyl and benzene ring signals indicates that PLRP may have interacted with polyphenols through hydrogen bonding and hydrophobic interactions.

**Figure 2:**
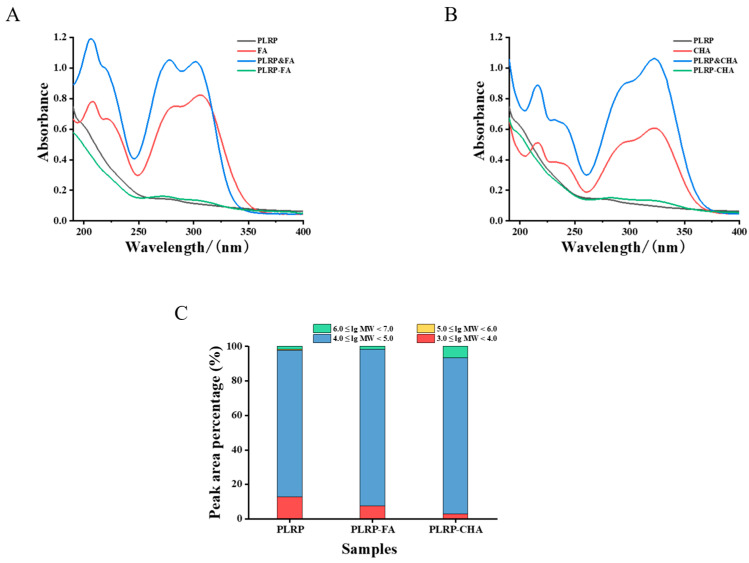
(**A**,**B**) UV-VIS spectra, (**C**) comparison of MW of the PLRP and PLRP-polyphenol complex.

As most polysaccharides do not have absorption peaks between 240 and 380 nm, the successful binding of polyphenols to polysaccharides can be confirmed by comparing the UV spectra of the polysaccharides, polyphenols, and their corresponding complexes [24]. Zhang et al. [25] found that FA was successfully adsorbed by pectin polysaccharides rich in arabinose, as confirmed by the corresponding UV and IR spectra. The formation of non-covalent complexes between polysaccharides and polyphenols can result in the weakening or disappearance of UV absorption peaks attributable to the polyphenols [26]. As shown in Figure 2A,B, the characteristic absorption peaks at 218, 288, and 312 nm near the PLRP&FA and PLRP&CHA mixtures and free polyphenols were significantly lower than those of PLRP-FA. Similarly, the characteristic CHA peaks in the UV spectrum of PLRP-CHA were also weakened, similar to previous studies. These data suggested that PLRP-FA and PLRP-CHA interacted and significantly changed the UV spectroscopic features of FA and CHA, confirming the molecular interactions between PLRP and polyphenols.

The Mw distributions of the PLRP-polyphenol complexes were determined via HPSEC-MALLS-RI analysis. The retention times and Mw of each fraction are shown in Table S2 and Figure S4, respectively. Compared with unpurified LRP, PLRP had fewer fractions (four), with an average Mw of 102.1 kDa. The RI chromatogram for PLRP primarily exhibited two peaks, indicating that Mw distribution was mainly in two fractions, 3.447 × 10^4^ (85.1%) and 5.660 × 10^3^ (12.6%), with the highest molecular weight reaching 4.187 × 10^3^ kDa (1.7%). In contrast, both the PLRP-FA and PLRP-CHA complexes had higher average Mw than PLRP, with Mw distributions mainly in two fractions, i.e., 3.728 × 10^4^ (75.5%) for PLRP-FA and 3.029 × 10^4^ (90.4%) for PLRP-CHA.

As shown in Figure 2C, the percentage of peak area for low-Mw fractions (3.0 ≤ lg Mw < 4.0) in PLRP was 12.6%, although it dropped to 7.3% for PLRP-FA and was undetected for PLRP-CHA complexes. The percentage of peak area for fractions with Mw in the range of 4.0 ≤ lg Mw < 5.0 significantly increased to 90.9% and 90.4% for PLRP-FA and PLRP-CHA. Additionally, both the PLRP-FA and PLRP-CHA complexes exhibited significantly higher Mw fractions (6.0 ≤ lg Mw < 7.0), indicating that the binding of phenolics to PLRP favored the formation of complexes with higher Mw and more compact conformations. Therefore, our findings suggested that PLRP formed hydrogen bonds with FA and CHA through non-covalent interactions.

### 3.2. Microstructural Characterization of PLRP-Polyphenol Complexes

**Figure 3:**
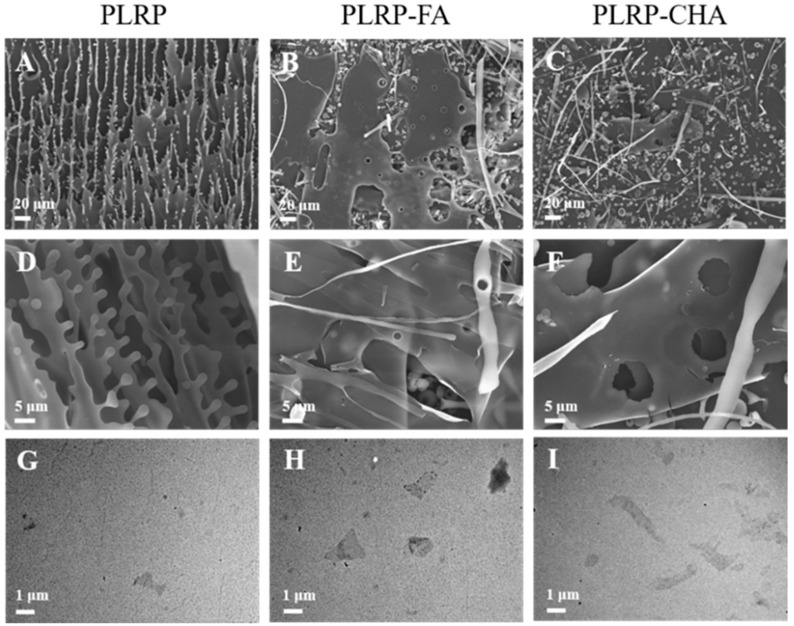
(**A**–**F**) SEM with different magnification of images of PLRP and PLRP-polyphenol complexes, (**G**–**I**) TEM images of PLRP and PLRP-polyphenol complexes.

As shown in Figure 3A–F, PLRP exhibited a regular layered structure with a uniform distribution, indicating the formation of a chain-like structure. As polyphenols exist in the form of needle-like crystals [27], when PLRP undergoes non-covalent binding with polyphenols, the size and shape of PLRP-polyphenol complexes differ significantly from the individual PLRP components. We observed that polyphenols self-assemble around PLRP and disrupt its morphology, causing the formation of cavities within PLRP. Previous data showed that surface hydroxyl groups on polysaccharides could form rigid structures with water, creating hydrophobic voids or gaps within the polysaccharide structure. Polyphenols could enter these voids or gaps through hydrophobic interactions [28]. This hydrophobic interaction was a key factor in polyphenol-polysaccharide binding, and hydrogen bonding could further enhance this binding effect. Similar research showed that after flavonoid adsorption, the Mw of corn silk polysaccharides increased, and the structure become more porous. After adsorption, the samples exhibited different morphological characteristics, with some visible particles (possibly flavonoids) aggregating on the polysaccharide surfaces. Binding-adsorption kinetics analysis revealed that higher-Mw polysaccharides formed more porous structures that were more conducive to flavonoid binding to polysaccharides [9].

Further microscopic observations of the morphology of the PLRP and PLRP-polyphenol complexes were conducted via TEM (Figure 3G–I). PLRP exhibited a uniform fibrous chain-like structure with minor clustering. However, the PLRP–polyphenol complexes showed an overall irregular clustered morphology with almost no linear structures. PLRP primarily adopted a linear conformation with limited hydrophobic cavities. PLRP mainly binds to polyphenols through hydrogen bonding, further enhancing the self-assembly of large molecules and resulting in a lamellar microstructure, consistent with our SEM analysis findings.

### 3.3. Immunomodulatory Activity of PLRP-Polyphenol Complex In Vitro

**Figure 4:**
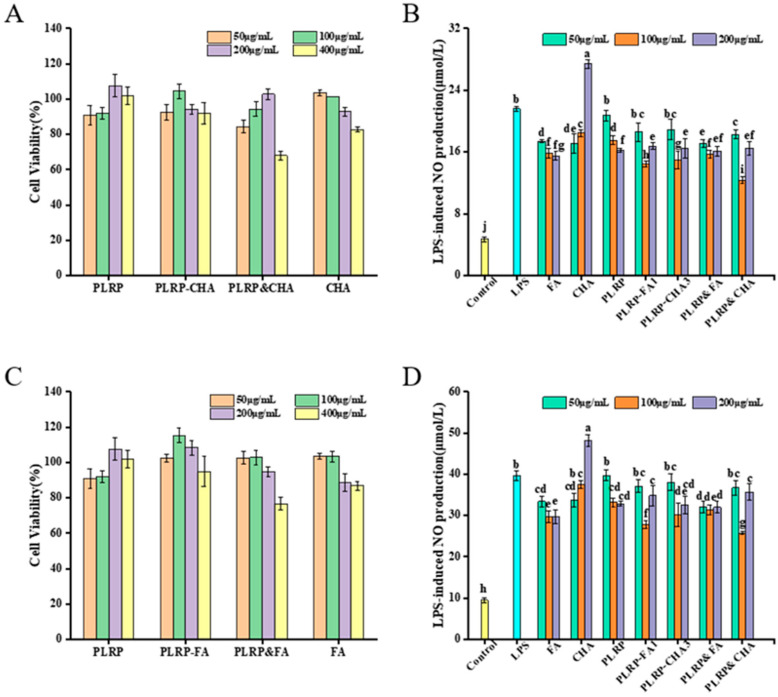
(**A**,**C**) Effect of PLRP and their complexes on the viabilities of macrophages. (**B**,**D**) The LPS-induced NO production of macrophages. (a–j indicates that there are significant differences between different letters, *p* < 0.05).

The impact of PLRP-polyphenol complexes on the viability of macrophage was assessed after exposing them to PLRP, the PLRP-FA, and PLRP-CHA (Figure 4A,C). Differing cell-viability rates were observed over the concentration range (400 μg mL^−1^) following treatment with PLRP-polyphenol complexes and their mixtures. Specifically, the cell viability of the PLRP&FA and PLRP&CHA groups decreased to 76.63% and 67.97%, respectively. However, at lower concentrations (50, 100, and 200 μg mL^−1^), the cell viabilities ranged from 82.75% to 115.41% (all exceeding 80%). Sample concentrations of 50, 100, and 200 μg mL^−1^ were chosen for subsequent experiments.

The influence of PLRP and its polyphenol complexes on NO production in LPS-induced macrophages was examined. We found stimulating macrophages with 200 μg mL^−1^ CHA (Figure 3B,D) exacerbated the LPS-induced inflammatory response. In contrast, PLRP and PLRP-polyphenol complexes (50 μg mL^−1^) did not significantly inhibit LPS-induced NO production (*p* > 0.05). However, treating macrophages with PLRP-polyphenol complexes and mixtures (100 μg mL^−1^) resulted in lower NO-production levels than PLRP treatment alone, all of which were lower than that in the LPS group (*p* < 0.05). Therefore, both PLRP and the PLRP-polyphenol complexes stimulated NO levels in macrophages to varying degrees and did not exacerbate the LPS-induced inflammatory response. Notably, no significant difference was found in NO production after treatment with the PLRP-polyphenol complexes and their corresponding mixtures [29]. These data suggested that PLRP-bound polyphenols and their mixtures could improve the inhibitory effect of PLRP and CHA on inflammation to some extent. However, non-covalent binding did not significantly affect the inhibition of NO production induced by LPS-induced inflammation in macrophages.

### 3.4. Impact of PLRP-Polyphenol Complexes on the In Vitro Antioxidant Activity of Macrophages

**Figure 5:**
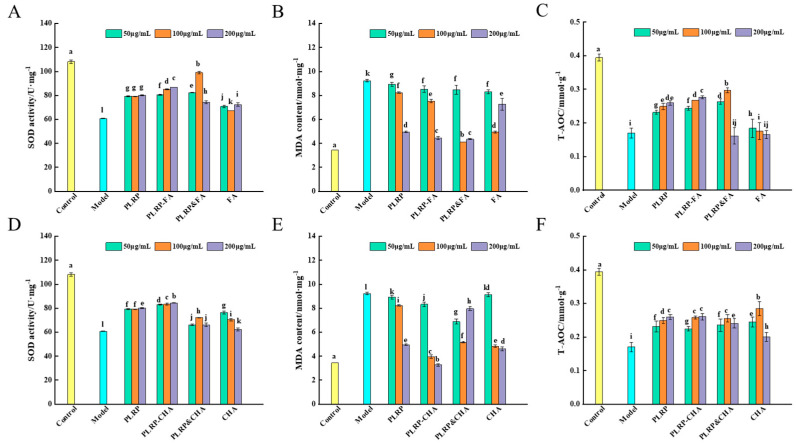
(**A**,**D**) Effects of PLRP-polyphenol complexes on SOD activity, (**B**,**E**) MDA activity, and (**C**,**F**) T−AOC activity in macrophages. (a–l indicates that there are significant differences between different letters, *p* < 0.05).

Figure S5 showed that the viability of macrophages significantly decreased with increasing H_2_O_2_ concentrations (*p* < 0.05) and H_2_O_2_ concentration (100 μmol L^−1^) that did not affect the normal growth of macrophages was selected. SOD efficiently scavenges free radicals within cells, protecting them from damage. Cellular SOD activities were significantly lower (*p* < 0.05) in the oxidative damage-model group than in the control group (Figure 5A,D). However, adding PLRP or PLRP-polyphenol complexes effectively enhanced the cellular SOD activities, with significant differences observed at different concentrations. The effects of the PLRP-polyphenol complexes were more prominent than those of PLRP alone (*p* < 0.05). Specifically, with the PLRP-CHA complex groups, all three dosages tested showed higher cellular SOD levels than the mixture and free CHA groups, indicating that non-covalent binding between PLRP and CHA enhanced the SOD activity in macrophages. Similar results were observed for the high-dosage group (200 μg mL^−1^) in terms of PLRP-FA complexes.

Furthermore, malondialdehyde (MDA) levels serve as a crucial indicator of organic lipid peroxide levels [30]. MDA levels serve as a crucial indicator of organic lipid peroxides levels. MDA levels not only reveal the extent of lipid peroxidation but also indirectly reflect the degree of membrane damage, providing insights into the extent of cell damage [30]. As shown in Figure 5B,E, MDA levels in the model group were significantly higher (*p* < 0.05) than those in the control group. The low-dosage groups (50 μg mL^−1^) of PLRP and PLRP-polyphenol complexes exhibited minimal differences when compared with those of the model group, although the medium and high-dosage groups (100 and 200 μg mL^−1^) significantly and dose-dependently suppressed MDA levels induction by H_2_O_2_ (*p* < 0.05). After treatment with 100 or 200 μg mL^−1^ PLRP-CHA complex group, MDA activities were significantly lower in cells treated with the corresponding mixture or free CHA (*p* < 0.05), thus reducing the extent of cell damage.

T-AOC helps to maintain the body in a relatively stable state by clearing excess ROS, ensuring a dynamic balance of ROS within the internal environment by preserving the overall antioxidant capacity of the body’s defense systems. As shown in Figure 5C,F, the cellular T-AOC activities of the model group were significantly lower (*p* < 0.05) than those of the control group. However, adding PLRP and PLRP-polyphenol complexes enhanced the cellular T-AOC activities of those in the damaged group (*p* < 0.05), and this enhancement exhibited a dose-dependent trend. Furthermore, the cellular T-AOC levels differed significantly in cells treated with PLRP-FA and PLRP-CHA complexes (200 μg mL^−1^) than in cells treated with the respective mixtures, free FA, and free CHA (*p* < 0.05).

### 3.5. RNA-Seq Analysis of Macrophages After PLRP-Polyphenol Complex Stimulation

RNA-seq was developed in the context of enhancing high-throughput sequencing [31,32]. After alignment, the average mapping rate of the samples to the reference genome was 90.15% and that to the reference gene set was 68.33%. In total, 16,497 genes were identified. The data quality is summarized in Table S3.

**Figure 6:**
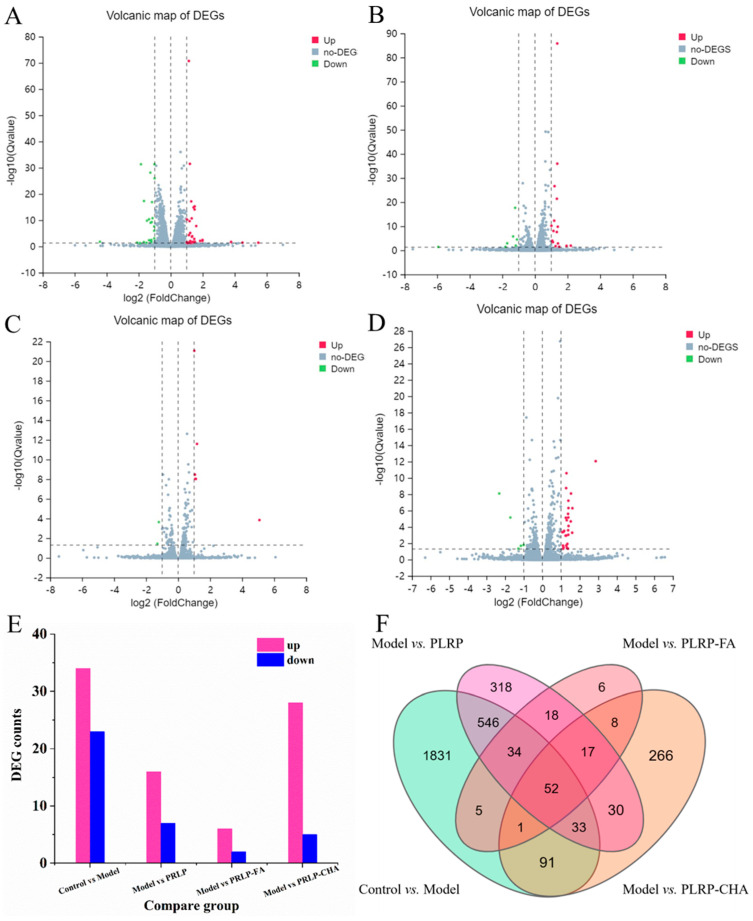
Volcanic and quantitative map of DEGs. (**A**) Control group and model group, (**B**) model group and PLRP group, (**C**) PLRP−FA group and (**D**) PLRP−CHA group. (**E**) Number of DEGs. (**F**) Venn analysis of DEGs.

The volcano plots are shown in Figure 6A–D presents the log2 FC on the X-axis and the negative logarithm (base 10) of the significance level on the Y-axis. DEGs were represented with red and blue dots, where the left side showed downregulated genes (relative to the control), the right side represented upregulated genes (relative to the control), and the gray region in the middle indicates genes that were not differentially expressed. We observed more expression of differential genes in the control and model groups. The PLRP-FA group showed relatively fewer expressions of differential genes, whereas the numbers of DEGs in the PLRP group and PLRP-CHA group were similar.

To further elucidate the extent of differential gene expression, using the transcripts-per-million method, the criteria for selecting DEGs were |log2 FC| ≥ 1 & *p* < 0.05. Compared with the control group, the model group had 57 DEGs, with 34 upregulated and 23 downregulated genes (Figure 6E). Compared with the model group, the PLRP group had 23 DEGs, with 16 upregulated and seven downregulated genes. The PLRP-FA group had eight DEGs, with six upregulated and two downregulated genes. The PLRP-CHA group had 33 DEGs, with 28 upregulated and five downregulated genes. Subsequently, we conducted an in-depth analysis of all the differentially expressed genes within the aforementioned four cohorts. The Venn diagram is shown in Figure 6F showed the differential gene intersection among four groups. The findings indicated that 52 genes exhibiting differential expression were prominently observed across the four groups, serving as the targeted gene sets for analysis.

**Figure 7:**
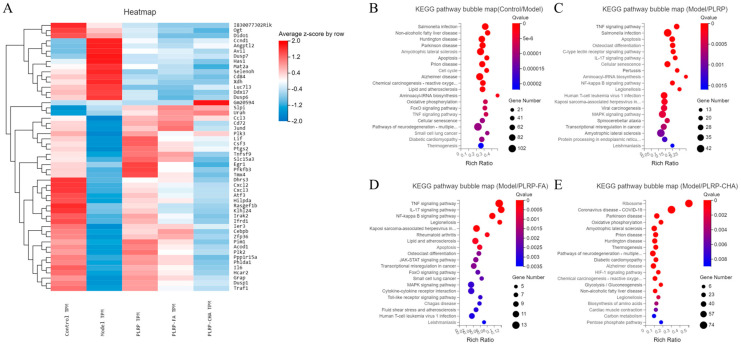
(**A**) Clustering heat map of DGEs. (**B**−**E**) KEGG Pathway enrichment bubble map analysis.

To further elucidate the specific expression patterns of significantly differentially expressed genes, clustering analysis was conducted on 52 selected target gene sets, and the cluster-analysis results were presented in Figure 7A. In comparison to the control group, the model group exhibited alterations following H_2_O_2_ stimulation, dehydrogenase/reductase 3, C-X-C motif chemokine ligand 2, and C-X-C motif chemokine ligand 3, RasGEF domain family, and kelch-like 24 were significantly downregulated. Studies revealed that dehydrogenase/reductase 3 was further examined for its function in retinoid metabolism, linked to antioxidants [33], and kelch-like 24 was correlated with intracellular oxidative stress. Meanwhile, Genes such as C-X-C motif chemokine ligand 2, C-X-C motif chemokine ligand 3 with RasGEF domain family have been identified to be implicated in the inflammatory process [34]. This occurs due to the oxidative stress damage induced by H_2_O_2_ in the model group, initiating an inflammatory response and resulting in the downregulation of these genes, signifying a decline in cell-specific function. Whereas cyclin D1, angiopoietin-like 2 and dual specificity phosphatase 7 were significantly upregulated. The upregulation of cyclin D1 is pivotal in the sustenance of cellular carcinogenesis and the acquisition of malignant characteristics [35]. Additionally, Angiopoietin-like 2 activated an inflammatory cascade in endothelial cells via integrin signaling and induced chemotaxis of monocytes/macrophages [36]. Moreso, relevant research indicated that dual specificity phosphatase 4 could be a viable target resistance inhibition and regulating the EMT during breast cancer treatment. In summary, the cells within the model group demonstrated a detrimental progression.

The genes of the PLRP group and PLRP-Polyphenol complex including 6-phosphofructo-2-kinase/fructose-2, 6-biphosphatase 3 and thioredoxin-related transmembrane Protein 4 were significantly downregulated compared to the model group. Among them, 6-phosphofructo-2-kinase/fructose-2 with 6-biphosphatase 3 is integrally involved in the regulatory control of glycolysis, which contributes to the cellular processes of expediting energy production in the absence of aerobic respiration [37]. Also, thioredoxin-related transmembrane Protein 4 is implicated in exerting a protective effect against inflammatory damage [38]. Especially, those genes in the PLRP-FA group included secretory leukocyte peptidase inhibitor, it has been documented that the abundance of gene expression is diminished amidst apoptotic alterations encountered by cells as they progress towards a cancerous state [39], and those in the PLRP-CHA group included mt-Rnr2 like 7 is associated with the protective effect of apoptosis induced by inflammation. Similarly, secretory leukocyte peptidase inhibitor genes in the PLRP-FA group exhibited a consistent upward trajectory. All these indicate the protective effect of polyphenols and their complexes on oxidative stress damage [40]. The expression levels of genes with cyclin D1 and angiopoietin-like 2 as well as dual specificity phosphatase 7 which plays a malignant role in cell growth were significantly lower in the PLRP, PLRP-FA, and PLRP-CHA groups, so this study fundamentally elucidates the beneficial impact of polyphenols and their complexes on modulating cellular activity within the experimental cohort.

To further delineate the characteristics of differentially expressed genes in macrophages, the GO database provides a comprehensive depiction of gene and gene-product properties in living organisms, including molecular functions (MF), cellular components (CC), and biological processes (BP). Figure S6 illustrated a representative GO-function set for each classification part. GO analysis of DEGs between the model group and control, or PLRP groups revealed 29 enriched BP terms, 18 CC terms, and 14 MF terms among the DEGs (Figure S6A,B). Analysis of the model group revealed that PLRP-FA and PLRP-CHA treatment resulted in enrichment for 26 and 28 BP terms, 15 and 18 CC terms, and 12 and13 MF terms among the DEGs, respectively (Figure S6C,D). The analytical outcomes indicated that, with the model group serving as the reference genome, the molecular functions of the differentially expressed genes in the experimental cohort were predominantly associated with enzymatic activity, molecular function modulation, and transcriptional control. Furthermore, the biological processes implicated primarily concerned metabolic pathways, biological regulation, and the upregulation of biological processes.

To further elucidate the function of DEGs, we conducted a KEGG functional annotation analysis for DEGs between the model and control groups, as well as between the PLRP and PLRP-complex treated groups. As depicted in Figure S6E–H, following H_2_O_2_ injury, DEGs in macrophages treated with PLRP or PLRP complexes exhibited relevant annotations for transport, catabolism, carbohydrate metabolism, nucleotide metabolism, and energy metabolism. Pathway-enrichment analysis helps provide a clearer understanding of the major biochemical metabolic pathways associated with DEGs and the signaling pathways connecting them. Figure 7B–E displayed bubble charts for the 20 most significantly enriched DEGs in typical pathways based on KEGG enrichment. KEGG pathway-enrichment analysis was performed on DEGs between the control and the model groups, revealing significantly enriched pathways, such as salmonella infection, amyotrophic lateral sclerosis, and hunting disease pathway. It was anticipated that the model group of cells would tend towards damage. Additionally, KEGG pathway-enrichment analysis was conducted on DEGs between the model group and the PLRP and PLRP-complex treated groups, showing significant enrichment in pathways such as the TNF, IL-17, NF-κB, and MAPK signaling pathways. These metabolic pathways are intricately associated with cellular self-repair mechanisms. Notably, the PLRP-complex cohort exhibits an absence of salmonella infection pathway compared to the PLRP cohort, suggesting that the remedial efficacy of the polyphenol complex surpasses that of conventional polyphenol groups. Furthermore, the model and PLRP polyphenol complex groups showed additional enrichment for pathways related to cytokine receptor interactions, the HIF-1 signaling pathway, and gluconeogenesis. Therefore, we speculated that during their responses to treatment, macrophages recognized pathogen/damage-associated molecular patterns through pattern recognition receptors on their cell membrane surfaces, subsequently activating downstream signaling pathways, including NF-κB and MAPKs.

## 4. Conclusions

In summary, PLRP exhibited a polysaccharide content of 90.08% and an average Mw of 102.1 kDa, and can assemble with phenolics by hydrogen bonding and hydrophobic interactions, resulting in non-covalent binding. The results of this study provided insights into the interactions between PLRP and polyphenolic compounds and their potential biological activities. Non-covalent binding between PLRP and phenolics led to the formation of high-molecular-weight complexes with compact conformations. These complexes exhibited promising antioxidant and anti-inflammatory effects on macrophages, and different doses of PLRP-FA and PLRP-CHA showed positive effects on damaged macrophages, especially at a high dose (200 μg mL^−1^), suggesting their potential use as functional food ingredients or nutraceuticals. The RNA-seq analysis further elucidated the underlying molecular mechanisms of PLRP and its complexes in modulating macrophage function, shedding light on their potential applications in immunomodulation and health promotion. Our findings suggest potential functional-food applications and therapeutic strategies for harnessing the benefits of polysaccharide-polyphenol interactions for enhanced immune and antioxidant response.

## Data Availability

The data that support the findings of this study are available in the Supplementary Materials of this article.

## Supplementary Materials

The following supporting information can be downloaded at: https://www.mdpi.com/article/10.3390/foods13223543/s1, Figure S1: Extraction flow chart of PLRP and PLRP-polyphenol complexe**s**; Figure S2: Fish-Bone Diagram depicting the various variables; Figure S3: Flowchart of RNA-seq test; Figure S4: HPSEC-MALLS-RI chromatogram of (A) PLRP, (B) PLRP-FA, (C) PLRP-CHA. Figure S5: Effects of different concentrations of H2O2 on proliferation of macrophages. Figure S6: (A–D) GO enrichment analysis of DEGs. (E–H) KEGG Pathway functional annotation analysis. Table S1: Preparation conditions of PLRP-FA and PLRP-CHA complexes; Table S2: The molecular weight distribution of PLRP, and phenolic complexes; Table S3; Data quality summary of RNA-seq.
