# Supplementary material for: Non-Covalent Interactions of Lotus Root Polysaccharides and Polyphenols and their Regulatory Mechanism on Macrophage Functions
Source: Foods. 2024 Nov 6;13(22):3543. doi: 10.3390/foods13223543 (PMC11592553; doi:10.3390/foods13223543)
Supplement: Supplementary file 1 [file foods-13-03543-s001.zip › foods-3277998-Supplementary Information.pdf]

Supplementary Information for

Non-Covalent Interactions of Lotus Root Polysaccharides and Polyphenols and their Regulatory Mechanism on Macrophage Functions

Yajie Li †, Nan Huang †, Qiulan Liu, Ying Sun, Kaidi Peng, Xueyu Jiang * and Yang Yi

Hubei Key Laboratory for Processing and Transformation of Agricultural Products, College of Food Science and Engineering, Wuhan Polytechnic University, Wuhan 430023, China; liyjie58@163.com (Y.L.); hyysgxnn@163.com (N.H.); liuqiulan575@163.com (Q.L.); sunying7535@163.com (Y.S.); kaidi19930218@outlook.com (K.P.); yiy86@whpu.edu.cn (Y.Y.)

* Correspondence: jiangxy@whpu.edu.cn

† These authors contributed equally to this work.

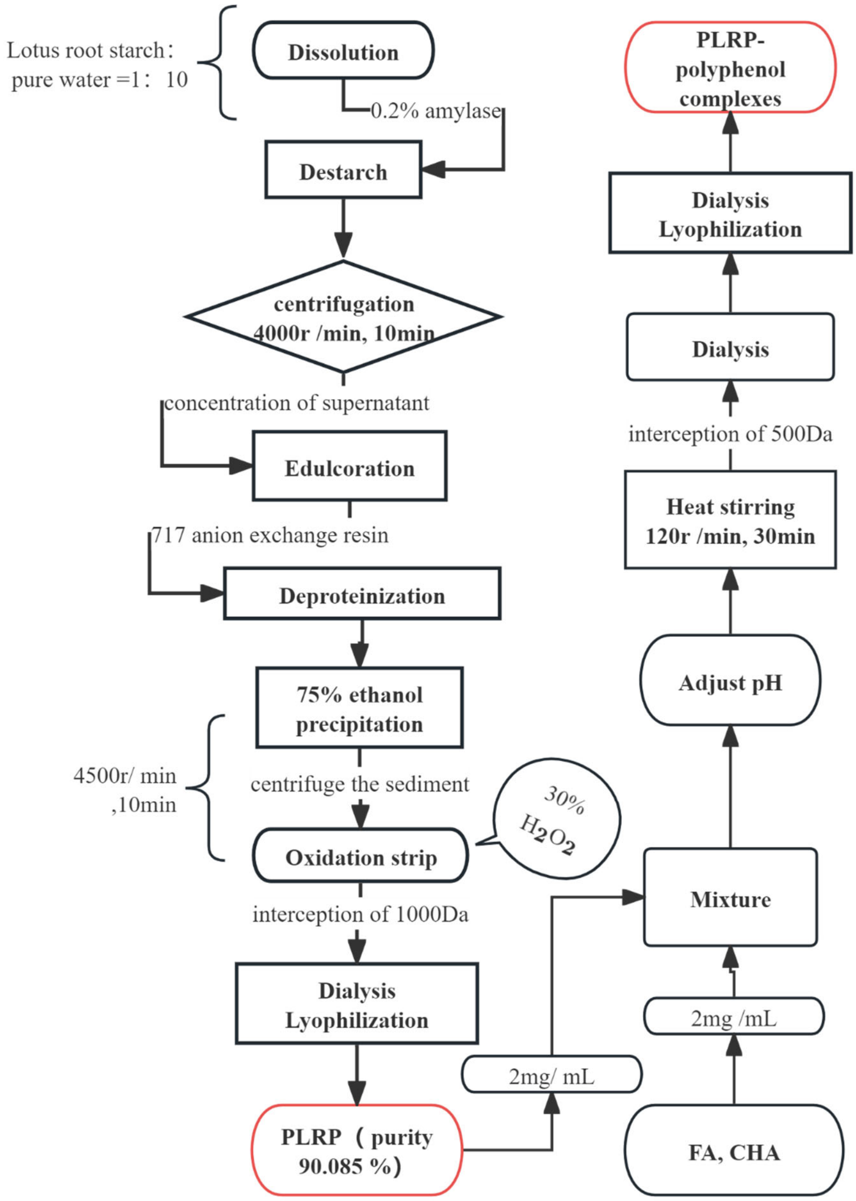

Figure S1. Extraction flow chart of PLRP and PLRP-polyphenol complexes.

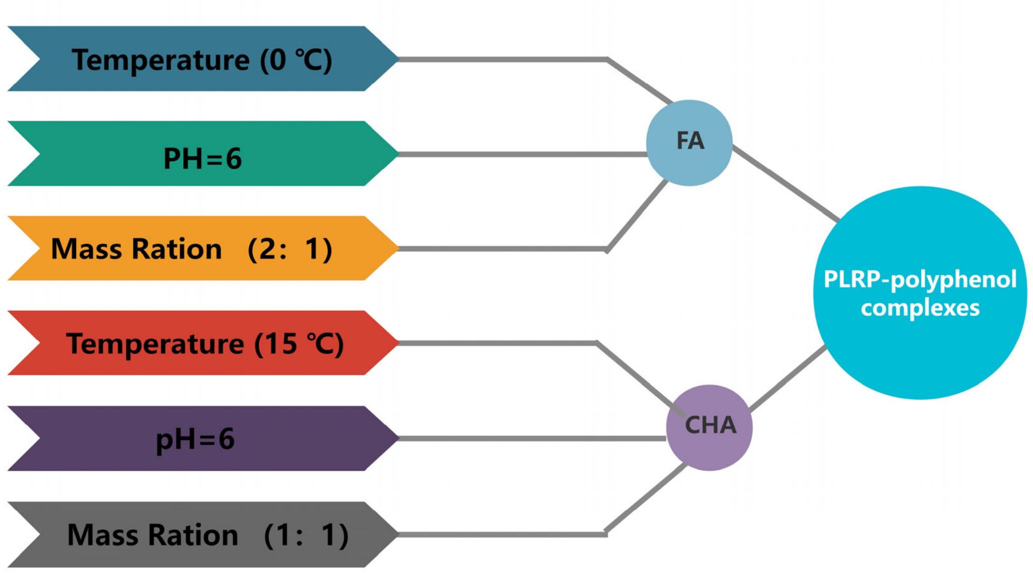

Figure S2. Fish-Bone Diagram depicting the various variables.

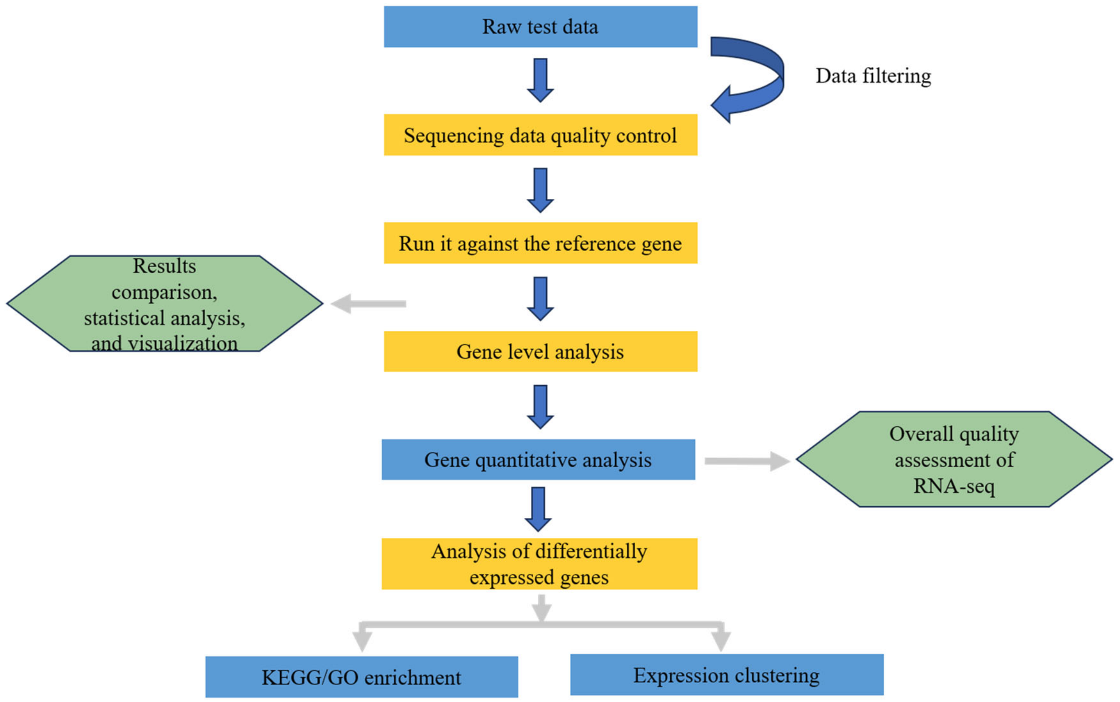

Figure S3. Flowchart of RNA-seq test.

Table S1. Preparation conditions of PLRP-FA and PLRP-CHA complexes.

Sample	Preparation condition		
	Temperature（℃）	pH	Mass ratio (polysaccharides: polyphenols)
PLRP-FA	0	6	2:1
PLRP-CHA	15	6	1:1

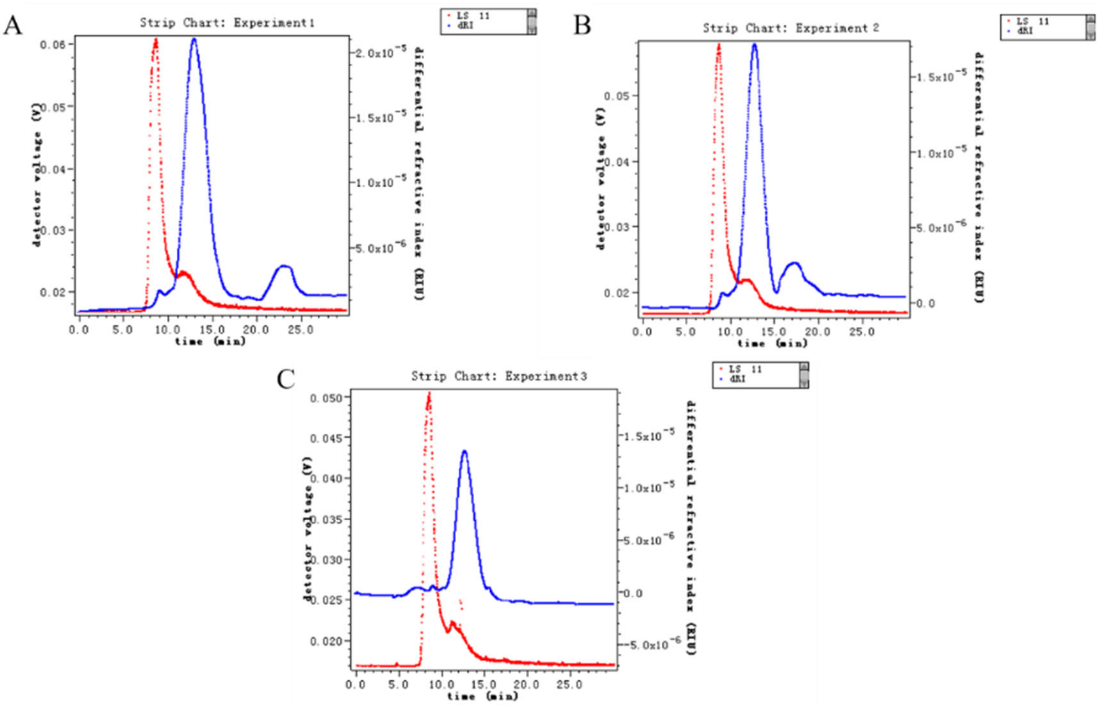

Figure S4. HPSEC-MALLS-RI chromatogram of (**A**) PLRP, (**B**) PLRP-FA, (**C**) PLRP-CHA.

Table S2. The molecular weight distribution of PLRP, and phenolic complexes.

Sample	Retention time（min）	Molecular weight（Da）	Content（%）	Average molecular weight（Da）
PLRP	7.274 - 9.718	4.187×10^{6} (±0.613%)	1.7	1.021×10^{5}
	9.718 - 17.810	3.447×10^{4} (±0.859%)	85.1	
	17.810 - 20.413	1.474×10^{5} (±3.646%)	0.6	
	20.413 - 25.840	5.660×10^{3} (±14.272%)	12.6	
PLRP-FA	7.627 - 9.787	3.890×10^{6} (±0.682%)	1.9	1.052×10^{5}
	9.787 - 15.153	3.728×10^{4} (±0.983%)	75.5	
	15.153 - 18.392	1.552×10^{4} (±2.897%)	15.4	
	18.392 - 21.186	9.815×10^{3} (±7.899%)	7.3	
PLRP-CHA	7.469 - 10.612	1.693×10^{6} (±0.687%)	6.7	1.434×10^{5}
	10.612 - 15.090	3.029×10^{4} (±1.354%)	90.4	
	15.090 - 17.535	8.914×10^{4} (±2.273%)	2.9	

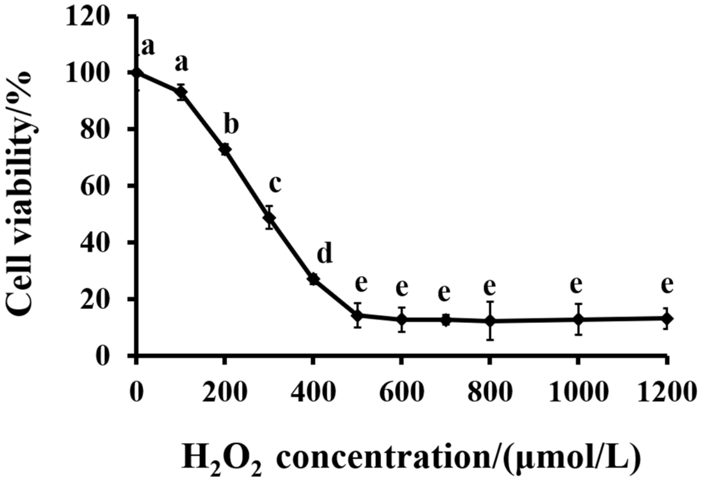

Figure S5. Effects of different concentrations of H_2O_2 on proliferation of macrophages.

Table S3. Data quality summary of RNA-seq.

Sample	Total Raw Reads (M)	Total Clean Reads (M)	Total Clean Bases (Gb)	Clean Reads Q20 (%)	Clean Reads Q30 (%)	Clean Reads Ratio (%)
Control_r1	48.28	42.86	6.43	97.29	91.28	88.78
Control_r2	49.48	43.60	6.54	97.53	92.02	88.12
Control_r3	50.68	43.96	6.59	97.59	92.22	86.74
Model_r1	52.43	47.54	7.13	96.31	88.97	90.68
Model_r2	48.93	43.99	6.60	97.52	91.96	89.90
Model_r3	50.68	44.17	6.63	96.62	90.22	87.16
PLRP_r1	48.93	43.93	6.59	97.54	92.03	89.77
PLRP_r2	48.93	44.58	6.69	97.54	92.01	91.10
PLRP_r3	50.29	43.64	6.55	97.72	92.59	86.79
PLRP-FA_r1	50.46	44.87	6.73	97.54	92.02	88.92
PLRP-FA_r2	50.68	44.75	6.71	97.56	92.11	88.29
PLRP-FA_r3	43.41	39.26	5.89	97.52	92.00	90.46
PLRP-CHA_r1	50.68	44.04	6.61	97.25	91.22	86.89
PLRP-CHA_r2	50.68	44.25	6.64	97.52	92.00	87.31
PLRP-CHA_r3	50.68	43.90	6.59	97.53	92.06	86.62

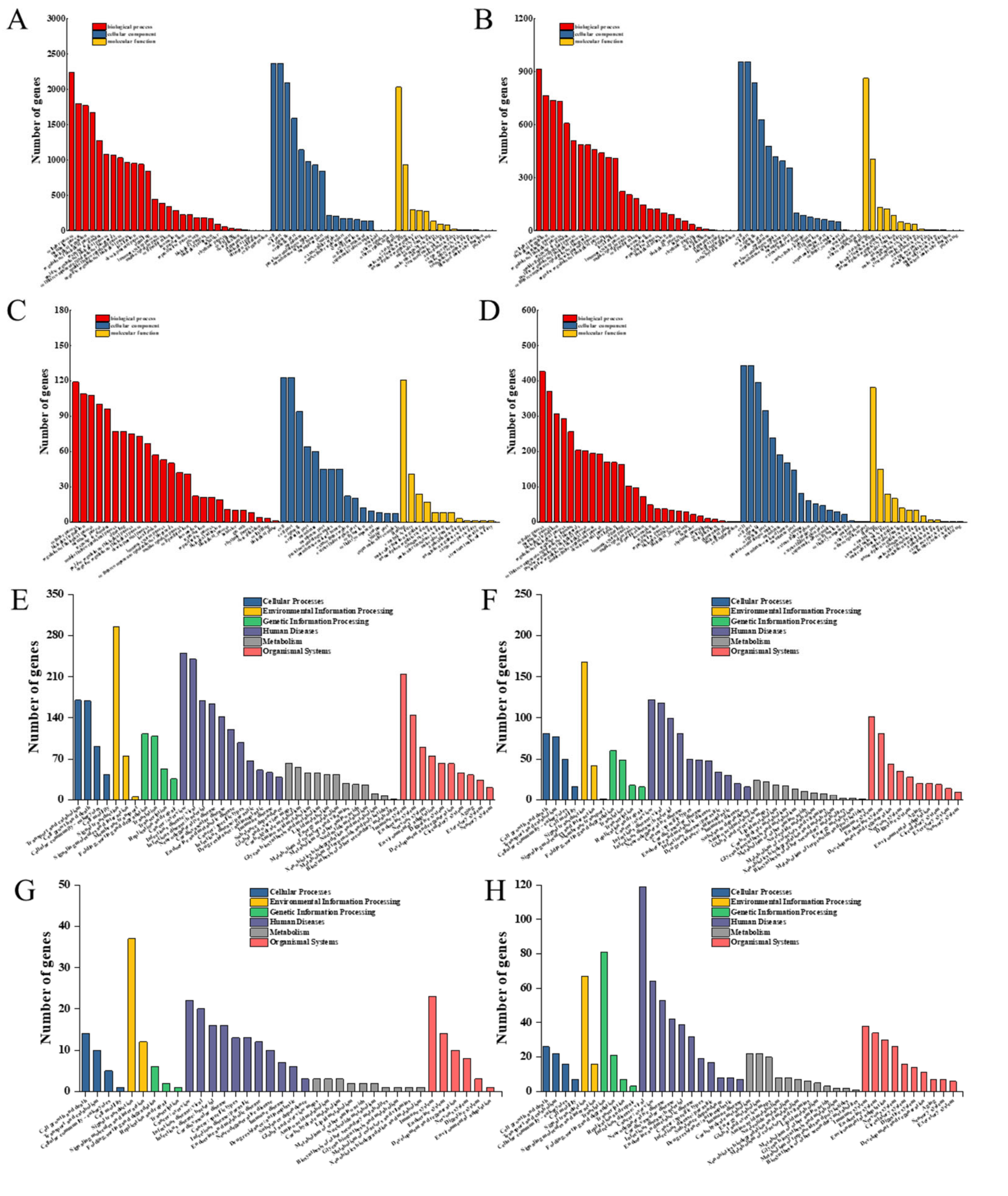

Figure S6. (**A-D**) GO enrichment analysis of DEGs. (**E-H**) KEGG Pathway functional annotation analysis.
